# Complete Genome Sequence of *Coprothermobacter proteolyticus* DSM 5265

**DOI:** 10.1128/genomeA.00470-14

**Published:** 2014-05-15

**Authors:** Alexandra Alexiev, David A. Coil, Jonathan H. Badger, Julie Enticknap, Naomi Ward, Frank T. Robb, Jonathan A. Eisen

**Affiliations:** a University of California Davis Genome Center, Davis, California, USA; b J. Craig Venter Institute, La Jolla, California, USA; c Cumnor House School, Danehill, West Sussex, United Kingdom; d University of Wyoming, Laramie, Wyoming, USA; e University of Maryland School of Medicine, Baltimore, Maryland, USA; f University of California Davis, Department of Evolution and Ecology, Department of Medical Microbiology and Immunology, Davis, California, USA

## Abstract

Here we present the complete 1,424,912-bp genome sequence of *Coprothermobacter proteolyticus* DSM 5265, isolated from a thermophilic digester fermenting tannery wastes and cattle manure.

## GENOME ANNOUNCEMENT

*Coprothermobacter proteolyticus* is a nonmotile, non-spore-forming, rod-shaped, Gram-negative anaerobic bacterium isolated from a thermophilic consortium fermenting tannery wastes and cattle manure (1). *C. proteolyticus* has increased utilization of fructose, mannose, glucose, maltose, and sucrose with the addition of yeast extract with either rumen fluid or Trypticase peptone compared to when it is grown without these additives (1). It was first considered a member of the genus *Thermobacteroides* but was latter reclassified as *Coprothermobacter proteolyticus* (2). *C. proteolyticus* was selected in 2002 as part of a National Science Foundation-funded “Assembling the Tree of Life” project at the Institute for Genomic Research (TIGR) to sequence the genomes of representatives of the seven phyla of bacteria that at the time had cultured representatives but no available genome sequence.

*C. proteolyticus* DSM 5265 was grown in DSM medium 481, and DNA was extracted using standard techniques. Sanger sequencing and genome assembly were performed as previously described for genomes sequenced by TIGR (3–5). Small and large insert plasmid libraries were constructed in pUC-derived vectors after random mechanical shearing (nebulization) of genomic DNA.

Sequencing resulted in 14,614 reads with an average read length of 1,039 bp and a coverage estimate of 10×. Sequences were assembled using Celera Assembler (6). The coverage criteria were that every position required at least double-clone coverage (or sequence from a PCR product amplified from genomic DNA) and either sequence from both strands or two different sequencing chemistries. The sequence was edited manually, and additional PCR and sequencing reactions were done to close gaps, improve coverage and resolve sequence ambiguities (7). All repeated DNA regions were verified by PCR amplification across the repeat and sequencing of the product. The full assembly consists of 1,424,912 bases and has a G+C content of 44.8%.

The replication origin was determined by colocalization of genes (*dnaA*, *dnaN*, *recF*, and *gyrA*) often found near the origin in prokaryotic genomes and G+C nucleotide skew (G·C/G+C) analysis (8). Completeness of the genome was assessed using the Phylosift software (9), which searches for 40 highly conserved, single copy marker genes (10). Thirty-nine of these 40 markers were found in this assembly and the missing marker (encoding porphobilinogen deaminase) was only found in 80% of the original 1,000 genomes used to generate the markers.

An initial set of open reading frames likely to encode proteins (coding sequences [CDSs]) were predicted as previously described (7). All predicted proteins larger than 30 amino acids were searched against a nonredundant protein database as previously described (7). Protein membrane-spanning domains were identified by TopPred (11). The 5′ regions of the CDSs were inspected to define initiation codons using similarity searches and to identify positions of ribosomal binding sites and transcriptional terminators. Two sets of hidden Markov models were used to determine CDS membership in families and superfamilies: Pfam v11.0 (12) and TIGRFAMs 3.0 (13). Pfam v11.0 hidden Markov models were also used with a constraint of a minimum of two hits to find repeated domains within proteins and mask them. This annotation was submitted with the genome in 2008, but in 2014 we requested an in-place update of the annotation from NCBI, using their integrated PGAP pipeline (14).

### Nucleotide sequence accession numbers.

This genome sequence has been deposited at DDBJ/EMBL/GenBank under the accession no. CP001145. The version described in this paper is version CP001145.1.

## References

[1] Ollivier BM Mah RA Ferguson TJ Boone DR Garcia JL Robinson R 1985 Emendation of the genus *Thermobacteroides*: *Thermobacteroides proteolyticus* sp. nov., a proteolytic acetogen from a methanogenic enrichment. Int. J. Syst. Bacteriol. 35:425–428. 10.1099/00207713-35-4-425

[2] Rainey FA Stackebrandt E 1993 Transfer of the type species of the genus *Thermobacteroides* to the genus *Thermoanaerobacter* as *Thermoanaerobacter acetoethylicus*(Ben-Bassat and Zeikus 1981) comb. nov., description of *Coprothermobacter* gen. nov., and reclassification of *Thermobacteroides proteolyticus* as *Coprothermobacter proteolyticus*(Ollivier et al. 1985) comb. nov. Int. J. Syst. Bacteriol. 43:857–859. 10.1099/00207713-43-4-857

[3] Wu D Daugherty SC Van Aken SE Pai GH Watkins KL Khouri H Tallon LJ Zaborsky JM Dunbar HE Tran PL Moran NA Eisen JA 2006 Metabolic complementarity and genomics of the dual bacterial symbiosis of sharpshooters. PLoS Biol. 4:e188. 10.1371/journal.pbio.0040188 16729848 PMC1472245

[4] Heidelberg JF Seshadri R Haveman SA Hemme CL Paulsen IT Kolonay JF Eisen JA Ward N Methe B Brinkac LM Daugherty SC Deboy RT Dodson RJ Durkin AS Madupu R Nelson WC Sullivan SA Fouts D Haft DH Selengut J Peterson JD Davidsen TM Zafar N Zhou L Radune D Dimitrov G Hance M Tran K Khouri H Gill J Utterback TR Feldblyum TV Wall JD Voordouw G Fraser CM 2004 The genome sequence of the anaerobic, sulfate-reducing bacterium *Desulfovibrio vulgaris* Hildenborough. Nat. Biotechnol. 22:554–559. 10.1038/nbt959 15077118

[5] Heidelberg JF Paulsen IT Nelson KE Gaidos EJ Nelson WC Read TD Eisen JA Seshadri R Ward N Methe B Clayton RA Meyer T Tsapin A Scott J Beanan M Brinkac L Daugherty S DeBoy RT Dodson RJ Durkin AS Haft DH Kolonay JF Madupu R Peterson JD Umayam LA White O Wolf AM Vamathevan J Weidman J Impraim M Lee K Berry K Lee C Mueller J Khouri H Gill J Utterback TR McDonald LA Feldblyum TV Smith HO Venter JC Nealson KH Fraser CM 2002 Genome sequence of the dissimilatory metal ion-reducing bacterium *Shewanella oneidensis*. Nat. Biotechnol. 20:1118–1123. 10.1038/nbt749 12368813

[6] Myers EW Sutton GG Delcher AL Dew IM Fasulo DP Flanigan MJ Kravitz SA Mobarry CM Reinert KH Remington KA Anson EL Bolanos RA Chou HH Jordan CM Halpern AL Lonardi S Beasley EM Brandon RC Chen L Dunn PJ Lai Z Liang Y Nusskern DR Zhan M Zhang Q Zheng X Rubin GM Adams MD Venter JC 2000 A whole-genome assembly of *Drosophila*. Science 287:2196–2204. 10.1126/science.287.5461.2196 10731133

[7] Tettelin H Radune D Kasif S Khouri H Salzberg SL 1999 Optimized multiplex PCR: efficiently closing a whole-genome shotgun sequencing project. Genomics 62:500–507. 10.1006/geno.1999.6048 10644449

[8] Lobry JR 1996 Asymmetric substitution patterns in the two DNA strands of bacteria. Mol. Biol. Evol. 13:660–665. 10.1093/oxfordjournals.molbev.a025626 8676740

[9] Darling AE Jospin G Lowe E Matsen FAt Bik HM Eisen JA 2014 PhyloSift: phylogenetic analysis of genomes and metagenomes. PeerJ 2:e243. 10.7717/peerj.243 24482762 PMC3897386

[10] Wu D Jospin G Eisen JA 2013 Systematic identification of gene families for use as “markers” for phylogenetic and phylogeny-driven ecological studies of bacteria and archaea and their major subgroups. PLoS One 8:e77033. 10.1371/journal.pone.0077033 24146954 PMC3798382

[11] Claros MG von Heijne G 1994 TopPred II: an improved software for membrane protein structure predictions. Comput. Appl. Biosci. 10:685–686 7704669 10.1093/bioinformatics/10.6.685

[12] Bateman A Birney E Durbin R Eddy SR Howe KL Sonnhammer EL 2000 The Pfam protein families database. Nucleic Acids Res. 28:263–266. 10.1093/nar/28.1.263 10592242 PMC102420

[13] Haft DH Loftus BJ Richardson DL Yang F Eisen JA Paulsen IT White O 2001 TIGRFAMs: a protein family resource for the functional identification of proteins. Nucleic Acids Res. 29:41–43. 10.1093/nar/29.1.41 11125044 PMC29844

[14] Angiuoli SV Gussman A Klimke W Cochrane G Field D Garrity G Kodira CD Kyrpides N Madupu R Markowitz V Tatusova T Thomson N White O 2008 Toward an online repository of Standard Operating Procedures (SOPs) for (meta)genomic annotation. Omics 12:137–141. 10.1089/omi.2008.0017 18416670 PMC3196215

